# Activating laboratory surveillance response to the threat of H5N1 influenza in the Veterans Health Administration, 2024–25

**DOI:** 10.3389/fpubh.2026.1869020

**Published:** 2026-07-30

**Authors:** Cynthia Lucero-Obusan, Joyce S. Chung, Gina Oda, Connor Edson, Aarthi Chary, Mark Holodniy

**Affiliations:** 1 Office of Public Health, US Department of Veterans Affairs, Veterans Health Administration, Washington, DC, United States; 2 National Public Health Reference Laboratory, US Department of Veterans Affairs, Veterans Health Administration, Palo Alto, CA, United States; 3 Division of Infectious Diseases and Geographic Medicine, Stanford University, Stanford, CA, United States

**Keywords:** avian influenza, H5N1 influenza, influenza surveillance, veterans health, whole genome sequencing (WGS), influenza a subtyping, epidemiology

## Abstract

**Introduction:**

The Veterans Health Administration (VHA) mobilized and adapted its established national infrastructure to enhance influenza surveillance during 2024–2025 highly pathogenic avian influenza (HPAI) A(H5N1) panzootic. Additionally, VHA's Public Health Reference Laboratory (PHRL) expanded capacity for H5 subtyping and whole genome sequencing (WGS) to support detection.

**Methods:**

Routine influenza surveillance was enhanced with customized queries to identify cases in high-risk counties with reported H5N1 detected in animals. In coordination with VHA clinical laboratories, select samples were sent to PHRL. PHRL subtyped specimens received, which included hospitalized cases. Demographics of influenza A cases in VHA were compared with those residing in high-risk “surveilled” counties (with reported H5N1 detection in animals) and those whose specimens were sent to PHRL for H5 subtyping.

**Results:**

From 4/1/2024-5/31/2025, we identified 40,406 positive influenza A VHA cases (12.5% with local subtyping performed), including 5,348 cases from “surveilled” counties. A total of 1,155 specimens were received by PHRL for H5 subtyping and 180 completed WGS. Cases from “surveilled” counties had fewer females, fewer in 50–69 age group and more of Hispanic ethnicity compared to other influenza A cases. No H5N1 was detected.

**Conclusion:**

We highlight the complexity and resources necessary for epidemiologic and laboratory surveillance of H5N1 within a large, national health system during a recognized panzootic. Additional resources for decentralized influenza A subtyping at local VA clinical laboratories are needed as centralized subtyping and WGS at PHRL was not comprehensive. Although H5 influenza has not yet been observed among Veterans within VHA, the ongoing risk warrants continued vigilance and monitoring.

## 1 Introduction

Influenza H5N1 (“bird flu”) is widespread in wild birds worldwide, resulting in spillover events and periodic outbreaks among other animals – including commercial poultry, backyard bird flocks, domesticated animals, wild terrestrial and marine mammals – and humans. A multistate panzootic of highly pathogenic avian influenza (HPAI) A(H5N1) in poultry, dairy cows and other animals began in the United States in the spring of 2024, involving at least 995 dairy herds in 17 states, 336 commercial poultry flocks and 207 backyard flocks across all 50 states with more than 90.9 million birds affected (1–5). Human cases in the U.S. were identified among individuals exposed to infected dairy cows and poultry (6–9). A total of 71 sporadic human cases of H5N1 influenza, at least 4 hospitalizations and two deaths were reported nationally as of March 2026, including 64 cases detected via targeted H5N1 surveillance of persons exposed to infected animals and seven cases through routine and enhanced influenza surveillance or clinician suspicion (10, 11). According to US Centers for Disease Control and Prevention (CDC) avian influenza A(H5N1) virus risk assessment, the current risk to the US general population remains low, although the dynamic situation and continued detection of human cases in other countries underscores the importance of proactive measures and continued vigilance (12).

Most influenza tests performed in clinical settings identify influenza A and B, but do not distinguish influenza H5N1 from H1 or H3 influenza. In the setting of this ongoing outbreak, tests positive for influenza A but negative for seasonal influenza A virus subtypes are prioritized for additional testing in a public health laboratory since few clinical or commercial laboratories offer H5 subtyping. Additionally, subtyping is recommended in those people meeting epidemiologic, clinical or public health response criteria, including people with a history of relevant exposure to influenza H5N1 virus-infected birds, dairy cows, persons or other animals as well as unprotected laboratory exposure (13).

The US Department of Veterans Affairs (VA), Veterans Health Administration (VHA) is the largest integrated healthcare system in the United States, with more than 7 million individuals treated annually at over 1,300 care sites located in 50 states, the District of Columbia (DC), and US territories (14). Veterans may be at higher risk for influenza and influenza complications, as they are older and have a higher burden of disease and comorbidities compared with the general US population (15, 16). VHA's Office of Public Health (OPH) analyzes electronic health record (EHR) data for seasonal influenza surveillance (17–20). OPH was requested to conduct enhanced surveillance for H5N1 influenza by the White House Office of Pandemic Preparedness in response to ongoing detections reported in humans and animals in the United States. Leveraging syndromic and laboratory data within this established infrastructure across the entire VHA network, we initiated enhanced, passive influenza surveillance to proactively monitor unusual trends and maintain situational awareness on influenza diagnoses within VHA. In addition, VHA's Public Health Reference Laboratory (PHRL) established capability to subtype H5 from respiratory and conjunctival samples in a cost-effective manner without the need for CDC confirmation. OPH, in collaboration with PHRL, had previously stood up the VA Sequencing for Research Clinical and Epidemiology (SeqFORCE) system as part of VA's COVID-19 response. SeqFORCE established a nationwide clinical and reference laboratory system for sequencing SARS-CoV-2, and expanded clinical operations, epidemiologic surveillance, and identification of this and other emerging pathogens (21, 22). We leveraged this system to increase capacity for influenza whole genome sequencing (WGS) within VHA.

VHA issued a clinical advisory in June 2024, followed by clinical guidance memorandum between January 8–13, 2025, containing guidance on novel influenza A(H5N1), including testing availability and criteria for testing. On January 16, 2025, CDC issued a Health Alert Network (HAN) advisory recommending subtyping of all influenza A virus-positive specimens from hospitalized patients on an accelerated basis to identify severe human infections with avian influenza A(H5) (23). In February 2025, PHRL sent a memo to all VA Medical Centers (VAMCs) with updated testing recommendations incorporating the accelerated subtyping recommendation. Facilities were encouraged to use a multiplex PCR assay that could differentiate H1 and H3 influenza A on specimens from anyone suspected of having H5N1 influenza based on clinical and/or epidemiologic risk factors and exposures as well as hospitalized patients, if they had the capability to do so. For specimens where subtyping could not or was not performed, or where sample results could not specify an H1 or H3 subtype at the local clinical laboratory, VA OPH encouraged these specimens be sent to PHRL for subtyping regardless of the patient's county of residence or exposure history.

The primary goal of this effort was to rapidly enhance and adapt the VA's influenza surveillance and laboratory testing infrastructure to proactively detect, monitor and characterize possible cases of H5N1 influenza A among Veterans. Our secondary objectives included: (1) refine VHA surveillance and testing strategies for novel influenza, using lessons learned from scaling up H5N1-specific monitoring; (2) evaluate the demographic, geographic and clinical characteristics of influenza A cases, including those from high-risk counties and hospitalized patients; (3) analyze laboratory findings, including distribution by clade/subclade and absence/presence of H5N1 in our cohort; (4) evaluate surveillance effectiveness to inform future public health response efforts by sharing insights and operational lessons.

## 2 Materials and methods

### 2.1 Epidemiologic surveillance of H5N1

OPH seasonal influenza surveillance methodology utilizes influenza-specific *International Classification of Diseases, Clinical Modification, 10*^*th*^ *Revision* (ICD-10-CM) coding and syndromic surveillance for healthcare encounters, including laboratory testing and pharmaceutical prescriptions extracted from the EHR as previously described (17). Enhanced H5N1 surveillance was developed using text-based triggers to alert for specified chief complaints including “bird flu”, “avian flu”, “conjunctivitis”, and “H5N1”, as well as for aberrations in clinical laboratory testing results for influenza A.

**Figure 1:**
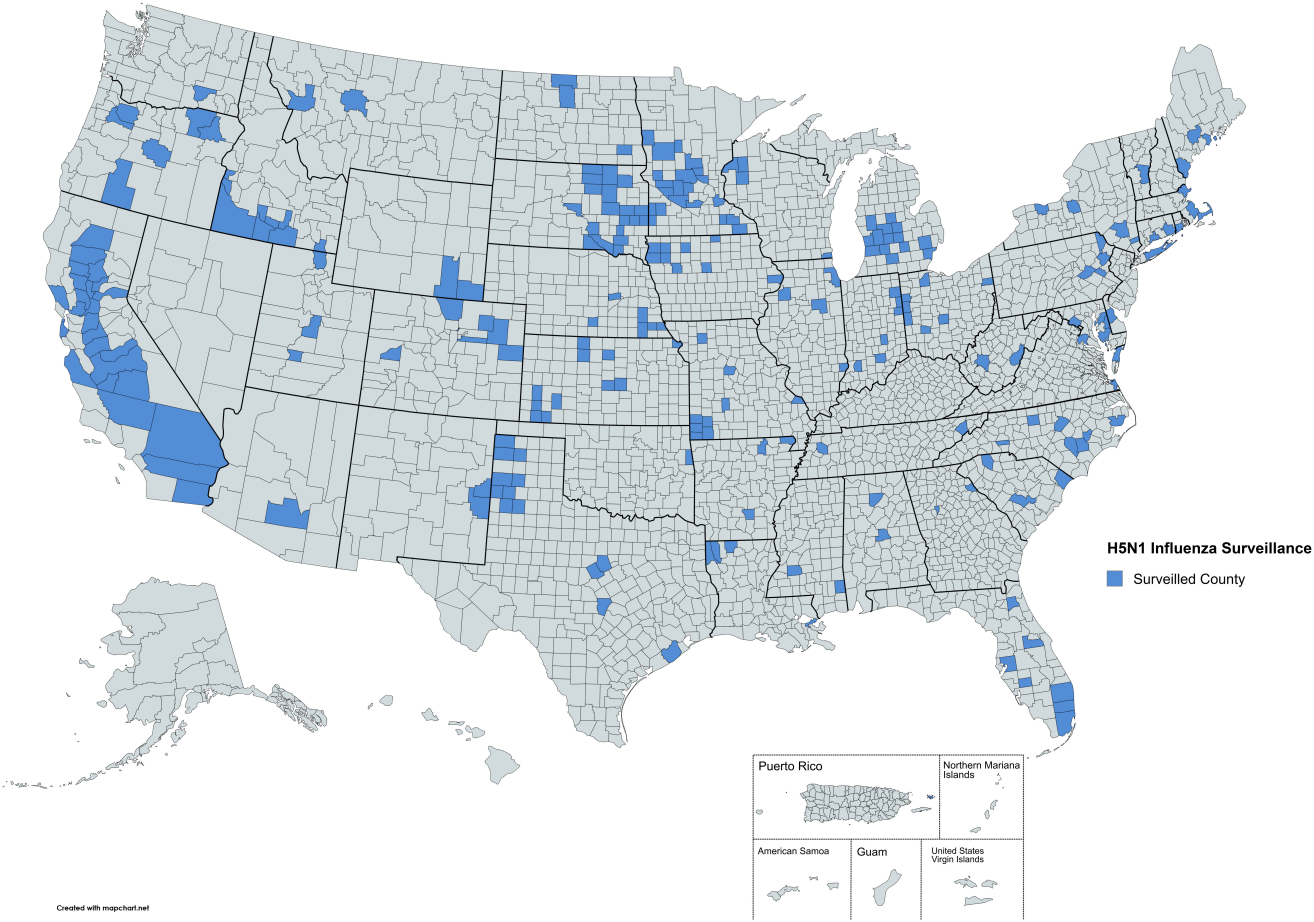
Veterans Affairs “Surveilled Counties” with confirmed H5N1 human, cattle, and/or poultry detections, April 2024–May 2025.

Presuming that geographic proximity increases the risk of possible H5N1 exposure from human or animal contact, including occupational hazards or other transmission routes, “surveilled counties” were identified and regularly updated. “Surveilled counties” included counties with confirmed positive H5N1 influenza human cases, or those with confirmed H5N1 found in cattle, livestock or commercial or backyard poultry flocks according to the US Department of Agriculture Animal and Plant Health Inspection Service, CDC, World Organisation for Animal Health or other publicly available sources (24–28) (Supplement material 1). There were 254 counties from 45 states classified as “surveilled counties” (Figure 1). The period of enhanced surveillance for these counties was at least 60 days after initial identification of animal or human H5N1 in each county. Surveillance continued beyond that for counties with human cases.

**Figure 2:**
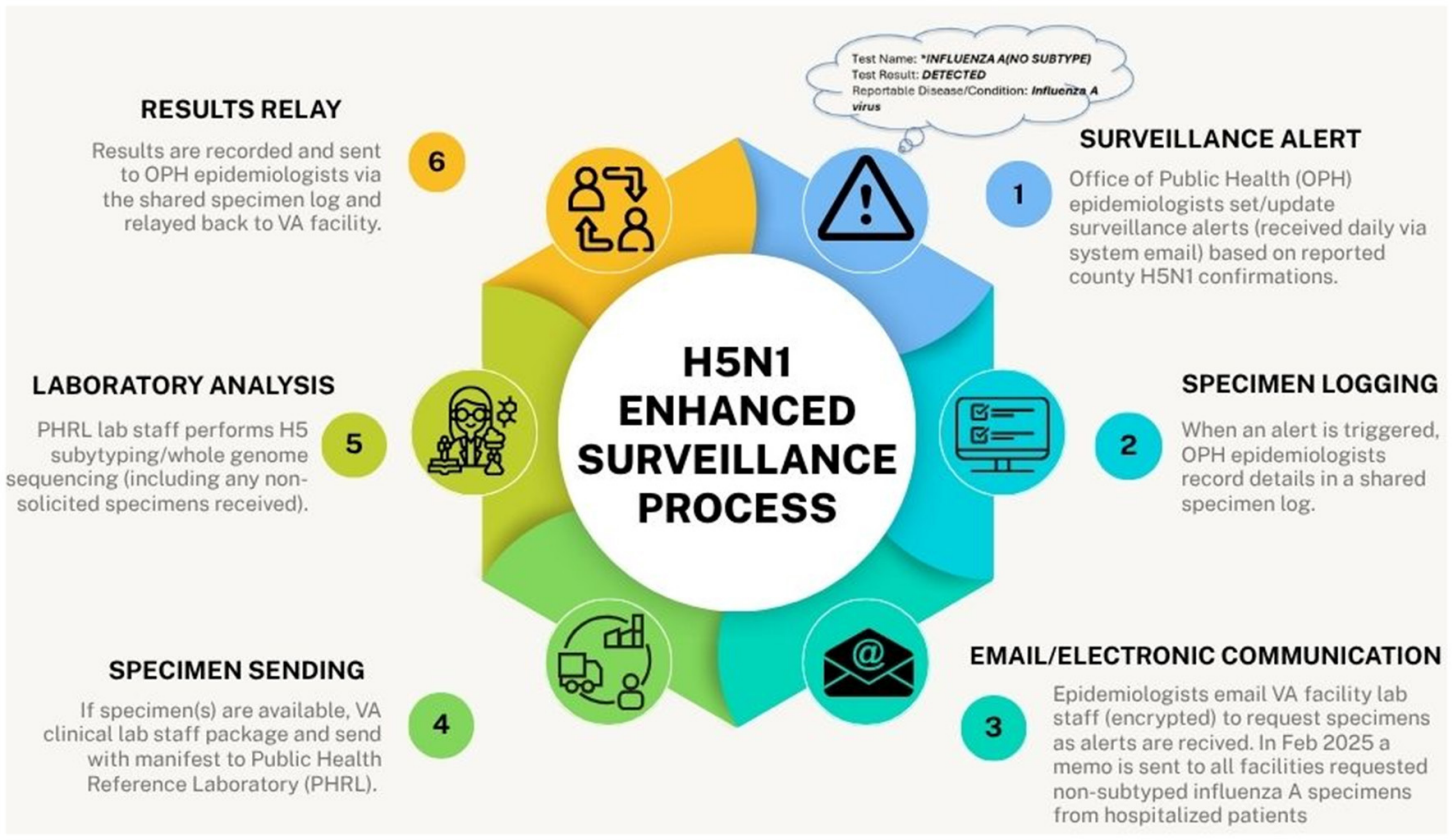
Veterans Affairs H5N1 surveillance and testing workflow, April 2024–May 2025.

Using the Praedico Public Health Surveillance System (Bitscopic) (29), alerts were then established to trigger when a patient residing in a surveilled county tested positive for influenza A and had either: an accompanying acute conjunctivitis (pink eye) diagnosis or the laboratory tests did not have a positive subtype test result of H1 or H3. During the period of enhanced surveillance, positive influenza A laboratory tests from patients residing in these surveilled counties were reviewed and VHA clinical laboratories received a single request to send the untyped specimens to PHRL for subtyping and possible sequencing (Figure 2). In addition, PHRL received specimens from hospitalized influenza A cases outside surveilled counties in response to the CDC HAN for accelerated subtyping for all hospitalized influenza A patients and as well as some unsolicited influenza specimens. Hospitalized cases were defined as specimens collected within 14 days prior to or any time during a recorded hospital admission.

### 2.2 Laboratory surveillance of H5N1

PHRL received and inventoried laboratory specimens from VHA clinical laboratories in response to OPH alert requests or the HAN/memo for subtyping of hospitalized influenza A specimens. Additionally, unsolicited influenza samples received from VAMCs across the network were also included. Instructions were given to VAMCs for local sample handling. Specimens could be refrigerated at 2–8 °C for up to 72 h. If the collection to receipt time was to exceed 72 h, the specimens were frozen at −70 °C and shipped on dry ice to PHRL. Samples were rejected if found to be thawed upon receipt at PHRL. Specimens that were positive or equivocal for influenza A at the local facility clinical laboratory were included in the PHRL cohort and analysis. Specimens from active-duty military and their dependents were excluded from our analysis. Specimens positive for influenza B or other respiratory pathogens were also excluded, unless the facility reported compatible symptoms with a relevant animal exposure and specifically requested H5 subtyping.

VAMC clinical laboratories used US FDA-approved nucleic acid extraction and real-time polymerase chain reaction (RT-PCR) assays, or integrated platforms used for influenza A/B sample testing of appropriate respiratory specimens. Respiratory (nasopharyngeal and nasal swab) and conjunctival samples meeting clinical/epidemiological criteria were sent to PHRL, where staff performed influenza subtyping confirmation and, when warranted, WGS to analyze virus clade and antiviral resistance. For speed and efficiency given the volume of samples received, PHRL bypassed H1/H3 testing and only performed Influenza A screening and H5 subtyping (CDC Human Influenza Real-Time RT-PCR Diagnostic Panel: Influenza A/B Typing Kit Influenza A/H5 Subtyping Kit, VER 4, CDC, Atlanta, GA Catalog # FluIVD03-12) according to the Instructions for Use. Briefly, influenza RNA was extracted from respiratory samples using the MagNA Pure 96 (Roche) and real-time RT-PCR (rRT-PCR) was performed using a 7,500 Fast Dx Real-Time PCR Instrument (Applied Biosystems). Cycle threshold (Ct) values from the influenza A screening RT-PCR assay were recorded. When all positive and negative controls exhibited the expected performance and the influenza A assay was less than 38.00 cycles, the specimen was considered positive for influenza. Samples where all controls exhibited the expected performance but no Ct value for influenza A was obtained were considered undetermined. Oligonucleotide primers and probes for characterization and differentiation of avian influenza A(H5) viruses from highly conserved regions of the hemagglutinin (HA) gene was used. The appropriate template file (Influenza A Subtyping Kit SuperScript) was selected and then results were analyzed for presumptive identification of influenza A and influenza subtype A(H5; Asian lineage) from viral RNA in human respiratory specimens.

Select samples underwent WGS using MagNA Pure 96 extracted RNA, followed by multi-segment reverse transcription-polymerase chain reaction (MRT-PCR) using universal primers for influenza A (30) followed by Nextera^®^ XT DNA library preparation (Illumina, San Diego, CA) to generate libraries for WGS on a MiSeq sequencer (Illumina) according to manufacturer instructions. Generated FASTQ files were subjected to a previously published analytic pipeline IRMA (Iterative Refinement Meta-Assembler) (31) and generated FASTA files were further analyzed using NextClade to determine viral clade and subclade type through hemagglutinin gene analysis (32). The FASTA sequences generated by the IRMA pipeline were submitted to the Global Initiative on Sharing All Influenza Data (GISAID) EpiFlu database (Supplement material 2) (33). WGS of influenza A-containing samples was conducted throughout the surveillance period as they were received. Samples were generally selected based on geography, specifically states where poultry, dairy cattle or human H5N1 infections were reported; had a Ct < 30 in our rRT-PCR influenza A assay; and additionally, could have had a Ct value > 31 or an undetermined Ct value if the sending site had documented a positive influenza A RT-PCR assay result. We also calculated median transit time from specimen collection to receipt at PHRL (excluding specimens with a missing collection date) and the turnaround time for processing specimens after receipt at PHRL (excluding specimens that required repeat subtyping).

### 2.3 Statistical methods

From these surveillance strategies, three distinct, non-overlapping groupings of influenza A cases (defined as unique VHA patients that tested positive for influenza A unduplicated within 30 days) were created: (1) overall VHA cases, (2) those residing in “surveilled counties”, and (3) those received by PHRL for H5 subtyping. Cases were categorized into groups in this order: group 3, group 2, group 1. Descriptive analysis of demographic, hospitalization status, and geographic characteristics of cases were analyzed, using Chi-square tests of homogeneity to compare the distributions between groups: (1 & 2), (2 & 3), and (1 & 3). Data analysis was conducted in RStudio version (2024.04.0 Build 735).

## 3 Results

### 3.1 Influenza a specimens

During the H5N1 surveillance period 4/1/24 to 5/31/2025, positive influenza A cases (*n* = 40,406) were identified from VHA jurisdictions including all 50 U.S. states, DC, and Puerto Rico. PHRL received 1,155 laboratory samples for subtyping for reasons noted above (group 3). One conjunctival specimen was received; the remaining specimens were from respiratory sources. Next, 5,348 influenza A cases were identified over the entire surveillance period from “surveilled counties” (group 2). The remaining 33,903 cases were classified as VHA (group 1).

**Figure 3:**
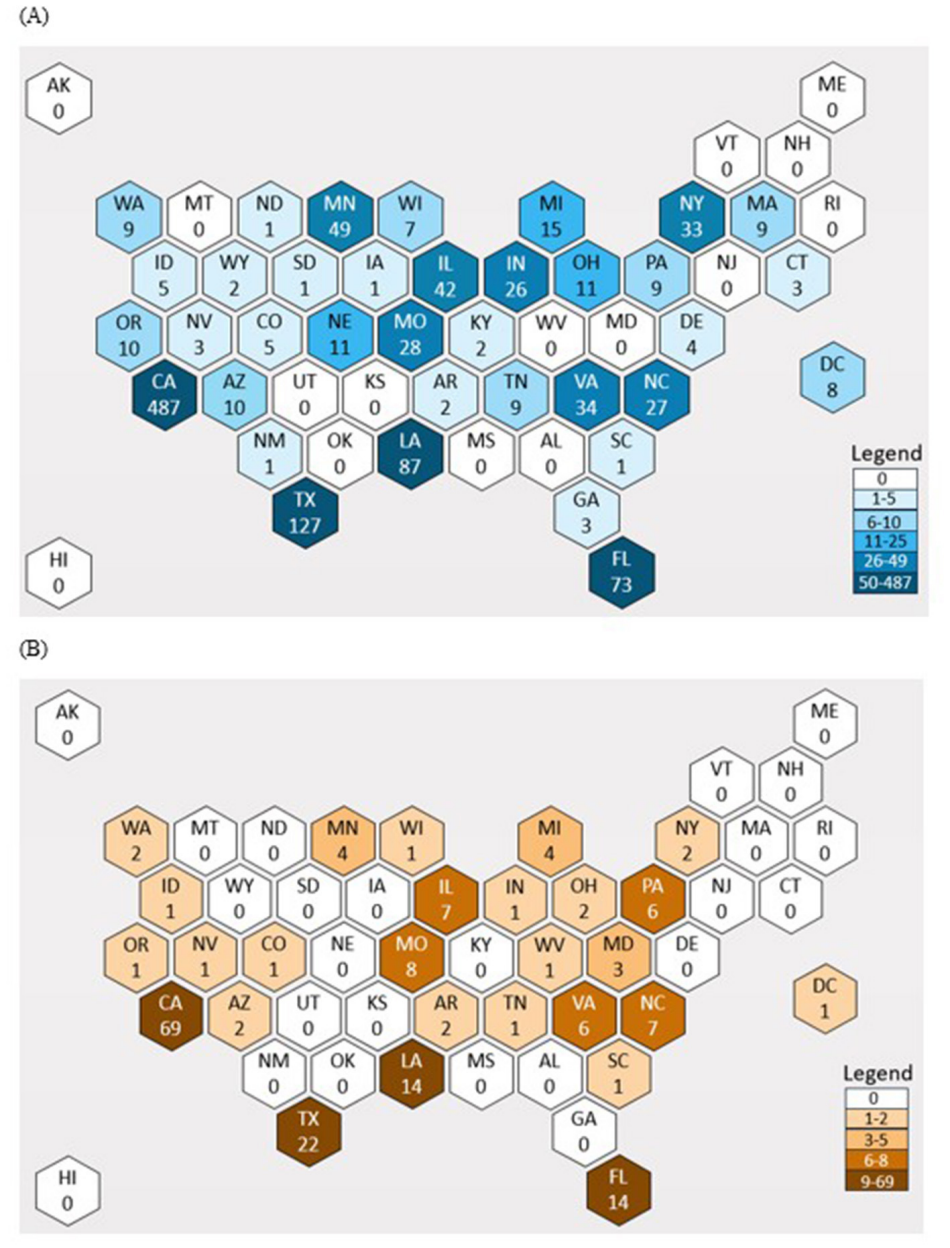
Distribution of influenza a laboratory specimens **(A)** and hospitalized influenza a laboratory specimens **(B)** received by Veterans Affairs Public Health Reference Laboratory, April 2024–May 2025.

**Figure 4:**
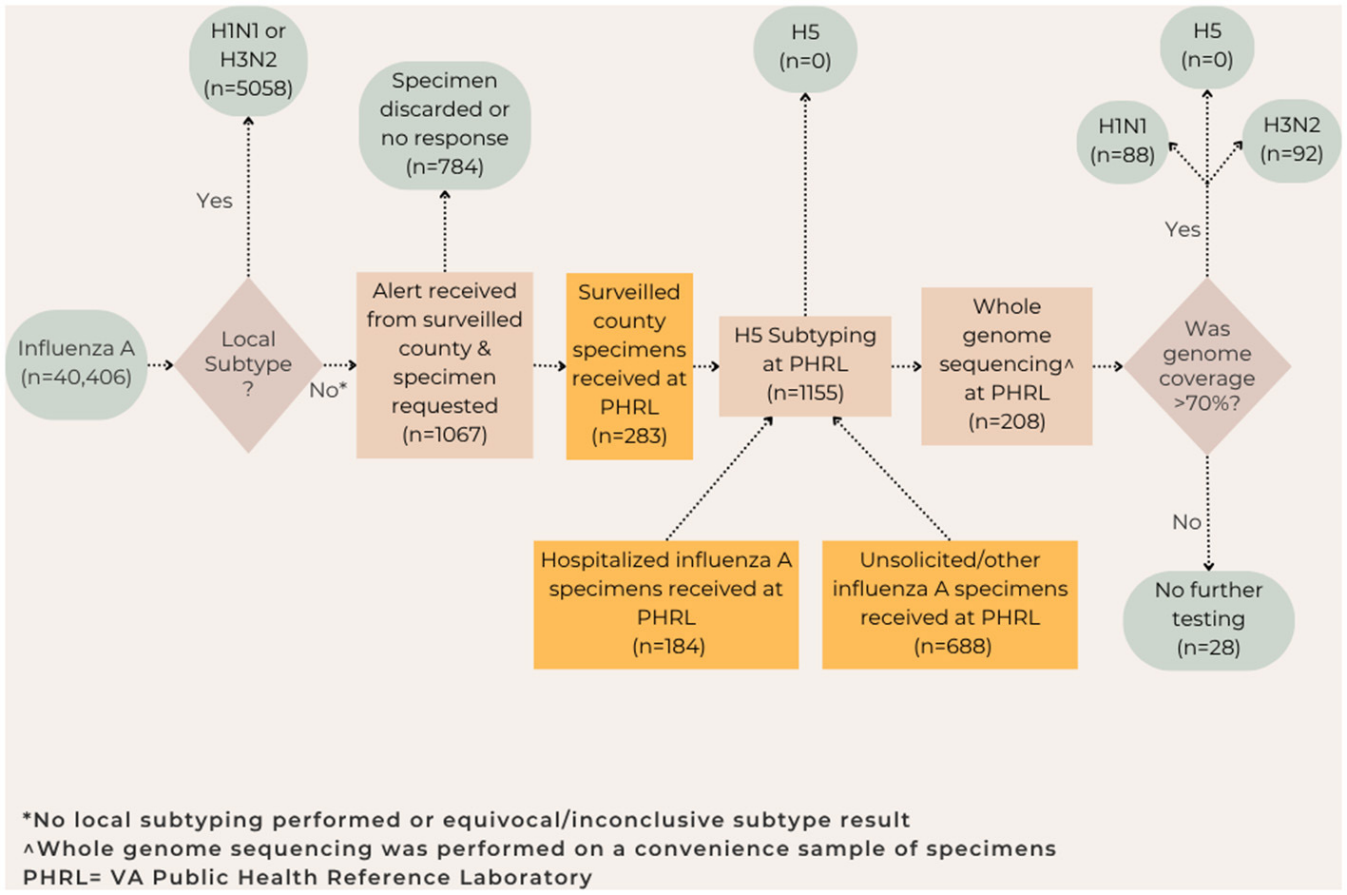
Flow diagram of influenza a specimens, Veterans Health Administration, April 2024–May 2025.

Among the group 3 PHRL received laboratory specimens, 642 (55%) were also determined to reside in “surveilled counties”. From the period of enhanced surveillance in the “surveilled counties”, PHRL received 283 samples from 73 facilities (26.5% response rate) from 1,067 requested. Laboratory specimens were submitted to PHRL from 36 states or jurisdictions (Figure 3A). Of samples received, 184 represented hospitalized cases, from 27 jurisdictions (Figure 3B); 141 (75%) of these samples were received after the January 2025 HAN/memo. A flowchart of specimens is presented in Figure 4.

### 3.2 Demographic analysis of positive influenza a cases

Table 1 displays the positive influenza A cases groups identified: (1) VHA cases (*n* = 33,903); (2) cases in “surveilled counties” (*n* = 5,348); and (3) cases with laboratory specimens sent to PHRL for H5 subtyping (*n* = 1,155). Overall, patients from the “surveilled counties” and those with laboratory specimens received for subtyping were similar. Compared to the cases from the larger VHA, the “surveilled” cases had notably higher Hispanic representation, fewer patients in the 50–69-year-old age group, and fewer females. Influenza vaccination status was similar between the groups. A modestly lower proportion of laboratory specimen cases were hospitalized compared to the other groups. Geographically, a high proportion of PHRL laboratory specimen cases were from the West (45.1%), with California accounting for the largest share.

**Table 1:** Characteristics of Positive Influenza A Cases for Veterans Health Administration system (1), Surveilled Counties (2), and Received Laboratory Specimens (3), April 2024–May 2025.

Characteristics	VHA^±^(1)		Surveilled Counties (2) ^†^		Lab Specimens (3)		*X*^2^ *p* (1, 2)	*X*^2^ *p* (1–3)	*X*^2^ *p* (2, 3)
	*n*	%	*n*	%	*n*	%			
Number of positive influenza A tests	33,903		5,348		1,155				
Age group									
< 29	1,369	4.0%	224	4.2%	55	4.8%	ns	ns	ns
30−49	8,095	23.9%	1,311	24.5%	265	22.9%	ns	ns	ns
50–69	13,610	40.1%	2,021	37.8%	440	38.1%	^**^	ns	ns
70+	10,829	31.9%	1,790	33.5%	395	34.2%	^*^	ns	ns
Sex									
Female	4,763	14.0%	674	12.6%	149	12.9%	^**^	ns	ns
Male	29,140	86.0%	4,674	87.4%	1,006	87.1%			
Race/Ethnicity									
White-non-Hispanic	17,866	52.7%	2,632	49.2%	548	47.4%	^***^	^***^	ns
Black-non-Hispanic	8,456	24.9%	1,316	24.6%	292	25.3%	ns	ns	ns
Asian-non-Hspanic	703	2.1%	134	2.5%	52	4.5%	^*^	^***^	^***^
American Indian or Alaska Native - non-Hispanic	322	0.9%	42	0.8%	13	1.1%	ns	ns	ns
Hispanic	3,420	10.1%	675	12.6%	137	11.9%	^***^	ns	ns
Unknown/Other	3,136	9.2%	547	10.2%	113	9.8%	^*^	ns	ns
Vaccination status									
Received vaccination during 2023–24 Influenza season	705	50.5%	116	45.1%	7	43.8%	ns	ns	ns
Cases during the 2023–24 Influenza season	1,396		257		16				
Received vaccination during the 2024–25 Influenza season	12,414	42.5%	1,844	43.8%	434	42.1%	ns	ns	ns
Cases during the 2024–25 Influenza season	29,204		4,209		1,031				
Unavailable	3,303		882		108				
Hospitalized	5,541	16.3%	866	16.2%	184	15.9%	ns	ns	ns
U.S. Regions									
West	5,673	16.7%	1,091	20.4%	521	45.1%	^***^	^***^	^***^
Midwest	6,815	20.1%	1,367	25.6%	198	17.1%	^***^	^***^	^***^
Northeast	2,533	7.5%	856	16.0%	51	4.4%	^***^	^***^	^***^
South	15,260	45.0%	1,768	33.1%	385	33.3%	^***^	^***^	ns
Other/Unknown	3,622	10.7%	266	5.0%	0	0.0%	^***^	^***^	^***^
States with most human and cattle/poultry cases									
California	2,529	7.5%	625	11.7%	467	40.4%	^***^	^***^	^***^
Colorado	397	1.2%	122	2.3%	4	0.3%	^***^	^*^	^*^
Washington	317	0.9%	26	0.5%	12	1.0%	^***^	ns	^*^
Subtyping results									
H1	2,502	7.4%	623	11.6%	21	1.8%	^***^	^***^	^***^
H3	1,402	4.1%	496	9.3%	14	1.2%	^***^	^***^	^***^
Untyped	29,999	88.5%	4,229	79.1%	1,120	97.0%	^***^	^***^	^***^

^*^p < 0.05, ^**^p < 0.01, ^***^p < 0.001, ns, Not Significant.

^±^Group 1 (VHA) represents influenza A, excluding surveilled counties (Group 2) and those specimens received for testing at PHRL (Group 3).

^†^Counties in which USDA and CDC had determined that dairy cattle and domestic poultry were infected with H5N1 or where human cases of H5N1 influenza were reported.

Some location information unavailable due to CDW/Oracle migration issues or in DC or US territories; Some unknown/missing demographic information is not presented.

### 3.3 Influenza a subtyping analysis

Influenza A subtyping was infrequently available in VAMC clinical laboratories (< 13% subtyped across entire VHA network, < 21% subtyped in “surveilled” counties). As OPH requested only untyped samples (i.e., specimens subtyped locally as H1 or H3 were not requested), nearly all laboratory specimens were untyped prior to receipt at PHRL (97%). The median Ct value was 26.67 (range 14.26-41.4, with 97 having an undetermined Ct value). Transit time from VAMC collection date to receipt at PHRL was a median of seven days (range 1–64 days). Turnaround time from PHRL receipt to H5 subtyping completed was a median of 6 days (range 0–57 days). No influenza A specimens were identified as H5N1 (Figure 4).

### 3.4 Whole genome sequencing results

Of the 1,155 influenza A specimens subtyped at PHRL, a convenience sample of 208 underwent WGS. Median Ct value of specimens undergoing WGS was 27.5 (range 14.26–41.03, with 23 having undetermined Ct value). Of the 208 Veteran samples attempted, 180 had >70% genome coverage (range 70.9–99.0%, median Ct value 27.26 with 15 having undetermined Ct value). As the WGS assay used different primers and conditions to enrich for influenza nucleic acid, we were successful in obtaining sufficient results to provide clade data from samples that had undetermined Ct values. Table 2 shows H1 and H3 subtype WGS results for selected specimens, representing 22 states plus the District of Columbia. Among states with at least 10 samples sequenced Texas and Minnesota samples predominantly identified as H3N2, Florida and Virginia predominantly H1N1, and California, Louisiana and Oregon with near-equal distributions of H1N1 and H3N2. Table 3 displays clade and subclade WGS results. H1N1 strains tested represented four clades, comprised of 14 subclades. H3N2 strains tested represented three clades and six subclades. Our data was consistent with clade/subclade analysis performed by CDC during the same time frame (34, 35). No sequenced samples were found to contain H5N1 strains, concordant with these samples' RT-PCR H5 subtyping results (Figure 4).

**Table 2:** Influenza whole genome sequencing of select specimens by state and subtype, Veterans Health Administration April 2024–May 2025.

State	H1N1	H3N2	Grand Total
Arizona	1	5	6
California (North)	14	10	24
California (Central)	-	3	3
California (South)	3	10	13
Colorado	2	-	2
Connecticut	1	-	1
District of Columbia	4	-	4
Florida	6	14	20
Idaho	-	1	1
Illinois	4	5	9
Indiana	1	1	2
Louisiana	7	5	12
Minnesota	12	2	14
Missouri	2	3	5
North Carolina	3	5	8
North Dakota	1	-	1
Nebraska	1	1	2
Nevada	1	1	2
New York	1	1	2
Ohio	4	2	6
Oregon	5	5	10
Pennsylvania	3	-	3
Texas	10	3	13
Virginia	2	13	15
Wyoming	-	2	2
Grand Total	88	92	180

**Table 3:** Influenza whole genome sequencing of select Specimens, clade/subclade results, Veterans Health Administration April 2024–May 2025.

Influenza virus subtype or lineage	HA clade	VHA # (% of subtype/lineage tested)	HA subclade	VHA # (% of subtype/lineage tested)
A/H1N1		88		88
	6B.1A.5a.2	9 (10%)	C	12 (14%)
	6B.1A.5a.2a	39 (44%)	C.1	6 (7%)
	6B.1A.5a.2a.1	38 (43%)	C.1.1	3 (3%)
	6B.1A.5a.2a.2	2 (2%)	C.1.9	15 (17%)
			C.1.9.1	3 (3%)
			C.1.9.2	1 (1%)
			C.1.9.3	14 (16%)
			C.1.9.4	1 (1%)
			D	4 (5%)
			D.1	1 (1%)
			D.3	19 (22%)
			D.3.1	4 (5%)
			D.4	1 (1%)
			D.5	4 (5%)
A/H3N2		92		92
	3C.2a1b.2a.2a	22 (24%)	G.1	22 (24%)
	3C.2a1b.2a.2a.3a	1 (1%)	G.1.3.1	1 (1%)
	3C.2a1b.2a.2a.3a.1	69 (75%)	J	4 (4%)
			J.2	61 (66%)
			J.2.2	2 (2%)
			J.2.5	2 (2%)

## 4 Discussion

### 4.1 Implications

In this study, we summarize H5N1 response efforts initiated in 2024 that leveraged and adapted VHA's existing surveillance infrastructure. Using enhanced passive surveillance and monitoring, molecular assays, and sequencing, we established a multipronged strategy to surveil for H5N1 influenza across the VHA network. H5N1 influenza was not detected through this comprehensive surveillance strategy; however, the possibility of undetected cases cannot be excluded. Given there is ongoing circulation of H5N1 in animal populations, a continued need for vigilance remains.

Although H5N1 influenza was not detected in this cohort, this study highlights the complexity, resources necessary and lessons learned from scaling surveillance for novel influenza detection within a national healthcare system. This can be beneficial for other health systems or jurisdictions wishing to scale and perform similar surveillance. Key insights from this experience help ensure surveillance integrity and can inform and guide refinement of future surveillance strategies within VHA as well as other healthcare and public health systems.

Foremost is the necessity of close partnership and communication between public health and clinical laboratories. The success of our process relied heavily on collaboration and coordination between OPH epidemiologists, PHRL laboratorians, and the network of medical technologists and lab managers across VAMC clinical laboratories.

Scaling up surveillance requires adequate resources for diagnostic testing, and expertise to perform specialized testing such as sequencing. That H5N1 influenza cases without documented animal or occupational exposure were reported by others, highlights the importance of agile point-of-care decentralized testing and subtyping capabilities across broad at-risk populations (11). This rationale supported our enhanced passive surveillance strategy focused on patients residing in counties where H5N1 influenza was detected in dairy cattle and poultry.

Notably, this strategy also hinged on robust and agile national common public health infrastructure and data sharing, specifically that of other public health entities testing for and reporting on animal and human H5N1 detections, and our own. Given the low (12.5%) percentage of influenza A specimens being subtyped at clinical laboratories in VHA, there was a risk of missing potential cases of H5N1 influenza. Since the VHA had an established and validated surveillance process already in place, we were well-positioned to adapt and target our passive surveillance efforts to counties at risk based on where the H5N1 detections were occurring. Because subtyping all influenza A specimens would have overwhelmed clinical laboratory systems, our strategy of targeting surveillance to higher risk geographic locations and centralized subtyping was more manageable and a better use of finite resources. Similarly, we were able to modify our passive surveillance to detect non-subtyped influenza A hospitalized cases in addition to our existing surveillance of counties with known H5N1 circulation.

Using targeted, enhanced surveillance we tagged approximately 4,700 untyped specimens in surveilled counties out of over 35,000 untyped specimens within VHA during this timeframe. Even with this targeted approach, the 26.5% response rate may have resulted in missed H5N1 influenza detection although the volume of samples received for subtyping at PHRL was still a significant challenge. Despite this, turn-around-time for H5 subtyping was typically within 6 days of receipt, depending on volume received and assays run per week. Many VAMCs had diagnostic assays that could only differentiate influenza A and B or did not routinely perform reflex subtyping. During the 2024−25 season, only 12.5% percent of all positive influenza A specimens in VHA were subtyped at VAMC laboratories. Based on clinical influenza testing from the past year, we found that 95 of 129 (74%) of VAMC parent laboratories reported H1N1 and H3N2 subtype results, indicating they can perform this test either in their clinical lab or as a send out test to a commercial laboratory. Subtyping was available in 83/102 (81%) of Level 1 (large, high complexity) and 2 (intermediate complexity) VAMCs but in only 12/27 (44%) of Level 3 (low or standard complexity) VAMCs. Moving forward, it will be important to strategically decentralize influenza A subtyping, by expanding H3/H1 subtyping capacity in VAMC clinical laboratories or developing other timely internal processes to automatically reflex positive influenza A results to a subtyping assay. Accordingly, PHRL would be able to focus its resources on H5 subtyping and WGS, which would be necessary for detecting and characterizing not only H5N1 but also other non-H3/H1 subtypes such as H5N5. We recognize that in the short term, particularly when test volume is high, there may not be capacity to subtype all influenza A specimens from hospitalized patients locally. Therefore, a hybrid risk-based approach for local vs. centralized subtyping may be needed, based on exposure history, clinical severity, or geographic proximity to animal/human cases. Additionally, we need to ensure testing availability and emphasize infection prevention and control measures, including influenza vaccination, appropriate personal protective equipment (PPE) and respiratory hygiene across our healthcare system.

### 4.2 Limitations

There are a few noted limitations regarding our VHA influenza surveillance experience with H5N1. Cases of H5N1 influenza could have been missed as not all individuals with influenza A at risk for H5N1 had subtyping or WGS performed, and those with mild illness may not have presented for care or had any testing performed. Some samples requested had already been discarded by the clinical laboratories. Most specimens collected were upper respiratory tract specimens with relatively few from lower respiratory tract or conjunctival specimens which may be preferred for suspected H5N1 infections (36). We also found that the patients' EHR had little/no exposure history regarding occupation, epidemiological links or risk factors for H5N1 exposure.

Though our process identified influenza specimens via a daily alerting mechanism, not all sample requests were successful as some samples were not available or able to be sent to PHRL. Moving forward, we may need to employ new strategies to improve response rate for specimen requests. Conversely, some sites sent samples for testing that were not requested. Nearly a third of laboratory specimens received by PHRL were from the West US region since a large proportion of counties in California had detection in dairy cattle. Furthermore, due to the large volume of specimens requiring subtyping, PHRL could not subtype and/or sequence all samples. As Ct values if available from the assay platform are not provided as part of the standard laboratory result in the electronic health record, we could not further discriminate which samples should be requested. Some of the samples had indeterminate influenza A results at PHRL although were positive for influenza A at the originating VAMC. We could not control sample storage or shipping from the originating site and so some samples could have degraded sufficiently to cause indeterminate results. However, some of these samples still yielded sufficient WGS coverage using a different assay format to confirm H5N1 was not detected. Enhanced influenza surveillance focused on county of residence and inpatient status as electronic data on occupation, epidemiological links or other risk factors for H5N1 exposure were not available. Although complex queries for automated detection were established, the process of recording and requesting specimens was a manual effort which was a rate-limiting step. Additional automation or development of a specimen dashboard could be beneficial future enhancements.

### 4.3 Conclusions

VHA worked to enhance surveillance and laboratory capacity for influenza H5 subtyping and sequencing in the wake of the US panzootic. Through this process, VA Public Health expanded virologic characterization of influenza cases while establishing a coordinated, efficient, epidemiologically sound and laboratory-informed approach to surveillance. Although H5N1 influenza has not been observed among Veterans within our health system, the ongoing risk warrants continued vigilance and monitoring. Although challenges in decentralizing testing remain, the framework of this approach could be implemented in future public health response efforts.

## Data Availability

The datasets presented in this study can be found in online repositories. The names of the repository/repositories and accession number(s) can be found in Supplementary Table 2 and at https://www.gisaid.org.
