# Supplementary material for: Activating laboratory surveillance response to the threat of H5N1 influenza in the Veterans Health Administration, 2024–25
Source: Front Public Health. 2026 Jul 30;14:1869020. doi: 10.3389/fpubh.2026.1869020 (PMC13468796; doi:10.3389/fpubh.2026.1869020)
Supplement: Supplementary file 1 [file Table_1.docx]

**Supplement 1. Process for identification and inclusion as a “surveilled county” formalized procedures in December 2024, Veterans Health Administration.**

Counties were identified by weekly review of the following primary data sources:

(1) US Department of Agriculture, *Confirmations of Highly Pathogenic Avian Influenza in Commercial and Backyard Flocks (*<https://www.aphis.usda.gov/livestock-poultry-disease/avian/avian-influenza/hpai-detections/commercial-backyard-flocks>). Data were reviewed for commercial products and WOAH poultry, and any new detections within the last 30 days.

(2) US Department of Agriculture *Detections of Highly Pathogenic Avian Influenza in Wild Birds. Updated November 12, 2025*. Available online at: <https://www.aphis.usda.gov/livestock-poultry-disease/avian/avian-influenza/hpai-detections/wild-birds>. Data were reviewed for any new wild bird detections within the last 30 days.

(3) US Department of Agriculture *Detections of Highly Pathogenic Avian Influenza in Mammals. Updated November 4, 2025*. Available online at: <https://www.aphis.usda.gov/livestock-poultry-disease/avian/avian-influenza/hpai-detections/mammals>. Data were reviewed for any new dairy or cattle detections within the last 30 days.

(4) World Organisation for Animal Health, World Animal Health Information System. *Animal Disease Events, Events management (*<https://wahis.woah.org/#/event-management>). Data were reviewed for counties with new outbreaks within any terrestrial mammals that were identified in the United States with High pathogenicity avian influenza virus (poultry)(inf with), subtype: H5N1.

(5) CDC and media reports of any human cases. Counties with human cases were not removed from our surveillance after identified.

**County list:**

| Chilton | Alabama |
| --- | --- |
| Cullman | Alabama |
| Pinal | Arizona |
| Clay | Arkansas |
| Cleveland | Arkansas |
| Sharp | Arkansas |
| Butte | California |
| Colusa | California |
| Fresno | California |
| Glenn | California |
| Imperial | California |
| Kern | California |
| Kings | California |
| Madera | California |
| Merced | California |
| Monterey | California |
| Placer | California |
| Riverside | California |
| Sacramento | California |
| San Bernardino | California |
| San Francisco | California |
| San Joaquin | California |
| San Mateo | California |
| Shasta | California |
| Solano, | California |
| Sonoma | California |
| Stanislaus | California |
| Sutter | California |
| Tehama | California |
| Tulare | California |
| Yolo | California |
| Yuba | California |
| Arapahoe | Colorado |
| Broomfield | Colorado |
| Delta | Colorado |
| Kit Carson | Colorado |
| Larimer | Colorado |
| Morgan | Colorado |
| Washington | Colorado |
| Weld | Colorado |
| New Haven | Connecticut |
| New London | Connecticut |
| Kent | Delaware |
| Broward | Florida |
| Clay | Florida |
| Desoto | Florida |
| Hillsborough | Florida |
| Miami-Dade | Florida |
| Orange | Florida |
| Palm Beach | Florida |
| Clayton | Georgia |
| Elbert | Georgia |
| Canyon | Idaho |
| Canyon | Idaho |
| Cassia | Idaho |
| Franklin | Idaho |
| Gooding | Idaho |
| Jerome | Idaho |
| Jerome | Idaho |
| Minidoka | Idaho |
| Owyhee | Idaho |
| Payette | Idaho |
| Twin Falls | Idaho |
| Cook | Illinois |
| Henry | Illinois |
| Lawrence | Illinois |
| Livingston | Illinois |
| Adams | Indiana |
| Allen | Indiana |
| Daviess | Indiana |
| Jackson | Indiana |
| Jay | Indiana |
| Shelby | Indiana |
| Cherokee | Iowa |
| Clinton | Iowa |
| Hamilton | Iowa |
| O'Brien | Iowa |
| Palo Alto | Iowa |
| Plymouth | Iowa |
| Sac | Iowa |
| Sioux | Iowa |
| Worth | Iowa |
| Doniphan | Kansas |
| Grant | Kansas |
| Gray | Kansas |
| Kearny | Kansas |
| Mcpherson | Kansas |
| Mitchell | Kansas |
| Phillips | Kansas |
| Rice | Kansas |
| Rooks | Kansas |
| Seward | Kansas |
| Stevens | Kansas |
| Bossier | Louisiana |
| Caddo | Louisiana |
| Claiborne | Louisiana |
| Orleans Parish | Louisiana |
| Kennebec | Maine |
| Knox | Maine |
| York | Maine |
| Caroline | Maryland |
| Charles | Maryland |
| Dorchester | Maryland |
| Montgomery | Maryland |
| Queen Anne's | Maryland |
| Barnstable | Massachusetts |
| Essex | Massachusetts |
| Plymouth | Massachusetts |
| Allegan | Michigan |
| Barry | Michigan |
| Calhoun | Michigan |
| Clinton | Michigan |
| Gratiot | Michigan |
| Ingham | Michigan |
| Ionia | Michigan |
| Isabella | Michigan |
| Kent | Michigan |
| Montcalm | Michigan |
| Newaygo | Michigan |
| Oakland | Michigan |
| Ottawa | Michigan |
| Wayne | Michigan |
| Benton | Minnesota |
| Chippewa | Minnesota |
| Clay | Minnesota |
| Dakota | Minnesota |
| Dodge | Minnesota |
| Fillmore | Minnesota |
| Kandiyohi | Minnesota |
| Lincoln | Minnesota |
| Lyon | Minnesota |
| Meeker | Minnesota |
| Morrison | Minnesota |
| Olmsted | Minnesota |
| Otter Tail | Minnesota |
| Pipestone | Minnesota |
| Renville | Minnesota |
| Sherburne | Minnesota |
| Sibley | Minnesota |
| Stearns | Minnesota |
| Stearns | Minnesota |
| Stevens | Minnesota |
| Swift | Minnesota |
| Wadena | Minnesota |
| Copiah | Mississippi |
| Greene | Mississippi |
| Barry | Missouri |
| Cooper | Missouri |
| Dallas | Missouri |
| Daviess | Missouri |
| Jasper | Missouri |
| Lawrence | Missouri |
| Mcdonald | Missouri |
| Newton | Missouri |
| Perry | Missouri |
| Cascade | Montana |
| Missoula | Montana |
| Gage | Nebraska |
| Johnson | Nebraska |
| Kearney | Nebraska |
| Lancaster | Nebraska |
| Nance | Nebraska |
| Nemaha | Nebraska |
| Sioux | Iowa |
| Curry | New Mexico |
| Roosevelt | New Mexico |
| Monroe | New York |
| Onondaga | New York |
| Putnam | New York |
| Suffolk | New York |
| Ulster | New York |
| Duplin | North Carolina |
| Guilford | North Carolina |
| Hyde | North Carolina |
| Lenoir | North Carolina |
| Lincoln | North Carolina |
| Sampson | North Carolina |
| Wake | North Carolina |
| Bottineau | North Dakota |
| McHenry | North Dakota |
| Ransom | North Dakota |
| Darke | Ohio |
| Franklin | Ohio |
| Greene | Ohio |
| Mahoning | Ohio |
| Mercer | Ohio |
| Van West | Ohio |
| Wood | Ohio |
| Adair | Oklahoma |
| Clackamas | Oregon |
| Crook | Oregon |
| Klamath | Oregon |
| Marion | Oregon |
| Umatilla | Oregon |
| Union | Oregon |
| Lehigh | Pennsylvania |
| Monroe | Pennsylvania |
| Schuylkill | Pennsylvania |
| Wayne | Pennsylvania |
| Culebra | Puerto Rico |
| Washington | Rhode Island |
| Horry | South Carolina |
| Orangeburg | South Carolina |
| Spartanburg | South Carolina |
| Beadle | South Dakota |
| Bon Homme | South Dakota |
| Brookings | South Dakota |
| Brown | South Dakota |
| Brule | South Dakota |
| Charles Mix | South Dakota |
| Day | South Dakota |
| Edmunds | South Dakota |
| Faulk | South Dakota |
| Gregory | South Dakota |
| Hanson | South Dakota |
| Hughes | South Dakota |
| Hutchinson | South Dakota |
| Kingsbury | South Dakota |
| Lake | South Dakota |
| Mcpherson | South Dakota |
| Miner | South Dakota |
| Moody | South Dakota |
| Spink | South Dakota |
| Gibson | Tennessee |
| Greene | Tennessee |
| Brazoria | Texas |
| Burnet | Texas |
| Castro | Texas |
| Comanche | Texas |
| Dallam | Texas |
| Deaf Smith | Texas |
| Erath | Texas |
| Hale | Texas |
| Hartley | Texas |
| Lamb | Texas |
| Moore | Texas |
| Parmer | Texas |
| Randall | Texas |
| Cache | Utah |
| Piute | Utah |
| Sanpete | Utah |
| Windsor | Vermont |
| Accomack | Virginia |
| Virginia Beach City | Virginia |
| Franklin | Washington |
| Kanawha | West Virginia |
| Pocahontas | West Virginia |
| Barron | Wisconsin |
| Burnett | Wisconsin |
| Kenosha | Wisconsin |
| Washburn | Wisconsin |
| Albany | Wyoming |
| Laramie | Wyoming |
