# Supplementary material for: Activating laboratory surveillance response to the threat of H5N1 influenza in the Veterans Health Administration, 2024–25
Source: Front Public Health. 2026 Jul 30;14:1869020. doi: 10.3389/fpubh.2026.1869020 (PMC13468796; doi:10.3389/fpubh.2026.1869020)
Supplement: Supplementary file 2 [file Table_2.docx]

Supplement 2. VHA FASTA sequences submitted to GISAID EpiFlu database (<https://www.gisaid.org>)

Accession IDs:

EPI_ISL_20376597

EPI_ISL_20379912

EPI_ISL_20379920

EPI_ISL_20379922-20379923

EPI_ISL_20379925

EPI_ISL_20379927

EPI_ISL_20379930-20379933

EPI_ISL_20379935-20379938

EPI_ISL_20379977-20380040

EPI_ISL_20379942- 20380043

EPI_ISL_20380045

EPI_ISL_20380048

EPI_ISL_20380054-20380062

EPI_ISL_20380065-20380068

EPI_ISL_20380070

EPI_ISL_20380074

EPI_ISL_20380076

EPI_ISL_20380078

EPI_ISL_20380080

EPI_ISL_20380082-20380102

EPI_ISL_20380106-20380110

EPI_ISL_20380112-20380114

EPI_ISL_20380116-20380122

EPI_ISL_20380125-20380130

EPI_ISL_20380132-20380133

EPI_ISL_20380135

EPI_ISL_20391744

EPI_ISL_20391750

EPI_ISL_20391975-20391978

EPI_ISL_20392006-20392013

EPI_ISL_20392045

EPI_ISL_ 20467723-20467724

EPI_ISL_20467726-20467740

EPI_ISL_20471145-20471146
